# Iron Nutrition and Its Biochemical Interactions in Plants: Iron Uptake, Biofortification, Bacteria, and Fungi in Focus

**DOI:** 10.3390/plants13050561

**Published:** 2024-02-20

**Authors:** Ferenc Fodor

**Affiliations:** Department of Plant Physiology and Molecular Plant Biology, ELTE Eötvös Loránd University, 1/C Pázmány Péter. sétány, H-1117 Budapest, Hungary; ferenc.fodor@ttk.elte.hu

Microelements are vital for plant growth and development. Among the micronutrients, iron is required in relatively high concentrations, and although it is one of the most abundant elements in the environment, its availability in soils is restricted due to its low solubility, especially in alkaline conditions and calcareous soils [1]. In order to cope with its limited availability, plants have evolved special mechanisms to mobilize, complex, reduce, and take up iron. It has been a long time since Römheld and Marschner [2] postulated the two basic strategies of iron uptake in higher plants. Strategy I is based on the reduction of soluble Fe-chelates in the apoplast by plasma-membrane-localised ferric reduction oxidase (FRO) family enzymes, which is followed by the uptake of ferrous ions by a divalent metal ion transporter of the iron-regulated transporter (IRT) family. This process is aided by several complementary mechanisms. Iron solubility is increased via the acidification of the rhizosphere by H^+^-ATPase enzymes located in the plasma membrane. In some plants, such as *Arabidopsis thaliana*, phenolics (mostly coumarines) are synthesized and secreted to the rhizosphere [3]. These may reduce and/or complex iron, thereby increasing its solubility. Other plants, such as *Cucumis sativus*, synthesize flavins (e.g., riboflavin) which increase iron availability by reduction and/or chelation or modifying the microbial population in the rhizosphere [4,5].

Strategy II is based on the synthesis and release of phytosiderophores (mugineic acids) by a transporter of mugineic acids (TOM). These compounds are strong iron chelators, mobilizing and chelating ferric iron in the soil; they are taken up without further chemical transformation by yellow stripe-like (YSL) transporters [6].

Strategy II was only described in graminaceous plants, whereas Strategy I was confined to the rest of higher plants. Nevertheless, combined strategies also occur, e.g., in *Oryza sativa*, both YSL and IRT1 proteins were found to facilitate iron uptake [7].

As a response to insufficient iron supply, the synthesis of proteins is upregulated in both strategies, accelerating the rate of iron uptake. After entering the cytoplasm, ferrous iron is thought to be complexed by glutathione (GSH) and/or nicotianamine; the latter is a ubiquitous amino acid derivative. Nicotianamine may have a key role in carrying iron to the sites of assimilation, storage, or retranslocation [8]. In the xylem, iron is translocated predominantly as Fe(III)-carboxylates such as Fe(III)3-(Citrate)3, which then become available in the apoplast for leaf cells [9].

The major sink of iron is photosynthetic apparatus. About 90% of tissue iron can be found in chloroplasts [10]. The mechanism of iron uptake to cellular compartments such as plastids, mitochondria, and the vacuole, has been the focus of more recent research, and despite the rapidly accumulating knowledge in this field, there are still unanswered questions. In chloroplasts and mitochondria, a reductive step by FRO enzymes seems to be necessary. Ferrous iron enters the stroma/matrix of these organelles, facilitated by another transport system: PIC1/TIC21 or nickel cobalt transporter (NiCo) in chloroplasts, and mitochondrial iron transporters (MITs) in mitochondria. In vacuoles, iron enters through vacuolar iron transporter (VIT) proteins; however, the reduction step has not yet been revealed [8].

The export of iron from the root and leaf cell cytoplasm as well as cell compartments are thought to be crucial in root-to-shoot transport, metabolic processes, loading of fruits and seeds and remobilization during senescence. Cellular export is facilitated by YSL family transporters but iron translocation in the xylem also relies on citrate loading to the xylem by ferric reductase defective 3 (FRD3) proteins. Iron export from chloroplasts may also occur through YSL proteins or a prokaryotic-type ECF ABC-transporter complex described recently [11]. Both chloroplasts and mitochondria release Fe-S proteins, which are important in cellular functions. Finally, iron is released from vacuoles through natural-resistance-associated macrophage proteins (NRAMP) [8].

Transport processes of iron may interact with divalent metal ions such as Cd, Mn, Ni, and Zn, but other elements may also influence iron metabolism. Competition or inhibition may occur in divalent transport systems, but also upon binding to phytosiderphores. Cadmium inhibits several metabolic processes in which it interacts with iron transport and assimilation. Iron acquisition in soils cannot be independent from other organisms such as fungi and bacteria. These may also release phytosiderophores or reduce iron, which can be highly advantageous for plants, especially under conditions of low iron availability. Iron deficiency in humans, known as anaemia, is one of the most critical micronutrient deficiencies worldwide; thus, it is a major goal to increase the bioavailable iron content in edible grains, fruits, and plant parts to serve as food products. Various techniques can be applied for the biofortification of iron, such as foliar sprays with Fe-chelates, iron-containing nanomaterials, or biostimulants; all these may influence iron uptake and assimilation processes. 

The published papers in this Special Issue focus on several aspects of iron nutrition and its interactions during uptake, transport, and assimilation. Increasing the bioavailability and uptake/accumulation of iron is still an important topic, as is iron incorporation into and release from cellular compartments. The presence of bacteria and fungi in the rhizosphere is under developing interest as iron acquisition by plants may be aided by these organisms. Although this Special Issue does not address the question of regulation of iron homeostasis and signalling, it is another very important area of current and future research that will help to develop a holistic picture on the metabolic roles and interactions of iron.
